# Tobacco Product Use Among Middle and High School Students — United States, 2020

**DOI:** 10.15585/mmwr.mm6950a1

**Published:** 2020-12-18

**Authors:** Andrea S. Gentzke, Teresa W. Wang, Ahmed Jamal, Eunice Park-Lee, Chunfeng Ren, Karen A. Cullen, Linda Neff

**Affiliations:** ^1^Office on Smoking and Health, National Center for Chronic Disease Prevention and Health Promotion, CDC; ^2^Center for Tobacco Products, Food and Drug Administration, Silver Spring, Maryland.

Tobacco use is the leading cause of preventable disease and death in the United States; nearly all tobacco product use begins during youth and young adulthood (*1*,*2*). CDC and the Food and Drug Administration (FDA) analyzed data from the 2019 and 2020 National Youth Tobacco Surveys (NYTS) to determine changes in the current (past 30-day) use of seven tobacco products among U.S. middle (grades 6–8) and high (grades 9–12) school students. In 2020, current use of any tobacco product was reported by 16.2% (4.47 million) of all students, including 23.6% (3.65 million) of high school and 6.7% (800,000) of middle school students. Electronic cigarettes (e-cigarettes) were the most commonly used tobacco product among high school (19.6%; 3.02 million) and middle school (4.7%; 550,000) students. From 2019 to 2020, decreases in current use of any tobacco product, any combustible tobacco product, multiple tobacco products, e-cigarettes, cigars, and smokeless tobacco occurred among high school and middle school students; these declines resulted in an estimated 1.73 million fewer current youth tobacco product users in 2020 than in 2019 (6.20 million) (*3*). From 2019 to 2020, no significant change occurred in the use of cigarettes, hookahs, pipe tobacco, or heated tobacco products. The comprehensive and sustained implementation of evidence-based tobacco control strategies at the national, state, and local levels, combined with tobacco product regulation by FDA, is warranted to help sustain this progress and to prevent and reduce all forms of tobacco product use among U.S. youths (*1*,*2*).

NYTS is a cross-sectional, voluntary, school-based, self-administered electronic survey of U.S. middle and high school students. A stratified three-stage cluster sampling procedure generated a nationally representative sample of U.S. students attending public and private schools in grades 6–12. Participants complete the survey in classrooms using a tablet computer.* In 2020, data collection occurred during January 16–March 16, 2020.^†^ In total, 14,531 students (participation rate = 87.4%) from 180 schools (participation rate = 49.9%) participated, yielding an overall response rate of 43.6% in 2020. Detailed information about NYTS is available elsewhere.^§^

Prevalence, with 95% confidence intervals, of current use of seven tobacco products (e-cigarettes, cigarettes, cigars, smokeless tobacco,^¶^ hookahs, pipe tobacco,** and heated tobacco products^††^) was reported; current use was defined as use on one or more days during the past 30 days. Three composite measures of current use (any tobacco product,^§§^ any combustible tobacco product,^¶¶^ and multiple tobacco products***) also were reported.

National weighted prevalence estimates and population totals^†††^ in 2020 were reported among all students and separately by school level. Estimates were reported overall and by selected demographic characteristics. Differences between the prevalence of current use in 2020 and that in 2019 (19,018 participants in 2019; student participation rate = 85.8%; school participation rate = 77.2%; overall response rate = 66.3%) were estimated using t-tests; p-values <0.05 were considered statistically significant. Trend analyses during 2011–2020 were not conducted because the mode of administration changed to an electronic survey in 2019 (*3*). The relative percent change (RPC) from 2019 to 2020 was calculated. Unstable estimates with a relative standard error of >30% or an unweighted denominator of <50 were suppressed. Analyses were conducted using SAS-callable SUDAAN (version 11.0.3; RTI International).

In 2020, among all students, 16.2% (an estimated 4.47 million) reported current use of any tobacco product (Table). Among high school students, 23.6% (3.65 million) reported current use of any tobacco product, 9.4% (1.45 million; 39.8% of any tobacco product users) reported current use of any combustible tobacco product, and 8.2% (1.27 million; 34.7% of any tobacco product users) reported current use of multiple tobacco products. By product, current use among high school students was highest for e-cigarettes (19.6%), followed by cigars (5.0%), cigarettes (4.6%), smokeless tobacco (3.1%), hookahs (2.7%), heated tobacco products (1.4%), and pipe tobacco (0.7%). Among high school students, any tobacco product use was reported by 24.7% of males and 22.5% of females; by 25.9% of non-Hispanic White, 23.3% of Hispanic, 18.4% of non-Hispanic Black, and 15.7% of non-Hispanic students of other races; and by 30.9% of those identifying as lesbian, gay, or bisexual, 22.0% of those identifying as heterosexual, and 20.4% of those reporting “not sure” about their sexual identity.

**TABLE:** Percentage of middle and high school students who reported current (past 30-day) tobacco product use, by product,* school level, sex, race/ethnicity, and sexual identity — National Youth Tobacco Survey, United States, 2020

Tobacco product	Sex		Race/Ethnicity				Sexual identity			Total	
	Female	Male	White, non-Hispanic	Black, non-Hispanic	Hispanic^†^	Other, non-Hispanic	Heterosexual	Lesbian, gay, bisexual	Not sure		
	% (95% CI)									% (95% CI)	Estimated weighted no.^§^
**Middle school and high school combined**											
E-cigarettes	12.7 (10.9–14.9)	13.4 (11.5–15.5)	15.5 (13.5–17.8)	6.2 (4.8–8.1)	13.7 (11.0–16.9)	7.7 (5.0–11.8)	12.3 (10.6–14.2)	20.2 (16.7–24.1)	7.5 (5.2–10.7)	**13.1(11.3–15.0)**	**3,580,000**
Cigars	3.4 (2.7–4.4)	3.7 (3.0–4.5)	2.8 (2.1–3.7)	6.5 (5.2–8.2)	4.0 (2.9–5.4)	—^¶^	3.1 (2.5–3.7)	6.0 (4.4–8.3)	3.0 (1.9–4.7)	**3.5 (2.9–4.3)**	**960,000**
Cigarettes	3.1 (2.4–4.0)	3.6 (2.7–4.7)	3.7 (2.8–4.8)	2.5 (1.8–3.5)	3.6 (2.6–4.9)	—^¶^	2.7 (2.1–3.6)	7.0 (5.1–9.4)	3.5 (2.2–5.5)	**3.3 (2.6–4.2)**	**910,000**
Smokeless tobacco	1.3 (0.9–1.7)	3.3 (2.5–4.3)	3.0 (2.3–3.9)	1.2 (0.6–2.1)	1.7 (1.3–2.2)	—^¶^	2.1 (1.6–2.8)	3.3 (2.2–4.8)	1.9 (1.1–3.3)	**2.3 (1.8–2.9)**	**630,000**
Hookahs	2.3 (1.7–3.0)	2.0 (1.6–2.5)	1.3 (1.0–1.7)	2.9 (2.1–4.0)	3.5 (2.5–5.0)	1.8 (1.0–3.1)	1.7 (1.4–2.1)	4.6 (3.4–6.1)	2.7 (1.5–4.7)	**2.1 (1.7–2.6)**	**580,000**
Heated tobacco products	1.4 (1.1–1.8)	1.3 (1.0–1.8)	1.1 (0.7–1.6)	1.1 (0.7–2.0)	2.1 (1.6–2.7)	—^¶^	1.0 (0.7–1.3)	3.2 (2.1–4.8)	—^¶^	**1.4 (1.1–1.7)**	**370,000**
Pipe tobacco	0.4 (0.3–0.6)	0.8 (0.5–1.1)	0.6 (0.4–1.0)	—^¶^	0.6 (0.4–0.9)	—^¶^	0.4 (0.3–0.7)	—^¶^	—^¶^	**0.6 (0.4–0.8)**	**150,000**
Any tobacco product**	15.8 (13.8–18.1)	16.7 (14.5–19.1)	17.8 (15.4–20.3)	13.2 (11.3–15.4)	17.2 (14.3–20.4)	10.1 (6.9–14.6)	15.1 (13.1–17.3)	25.5 (21.8–29.5)	11.1 (8.3–14.7)	**16.2 (14.3**–**18.4)**	**4,470,000**
Any combustible tobacco product^††^	6.6 (5.5–7.9)	7.0 (5.8–8.4)	5.9 (4.7–7.4)	9.2 (7.8–10.7)	8.1 (6.4–10.3)	4.9 (3.2–7.4)	5.7 (4.7–6.8)	13.5 (11.0–16.5)	6.9 (5.0–9.3)	**6.8 (5.8–7.9)**	**1,870,000**
Multiple tobacco products^§§^	5.3 (4.4–6.6)	6.5 (5.2–8.0)	6.1 (4.9–7.6)	4.9 (3.9–6.0)	6.7 (5.1–8.7)	4.3 (2.8–6.4)	5.0 (4.0–6.1)	11.7 (9.4–14.6)	5.6 (3.7–8.2)	**5.9 (4.9**–**7.1)**	**1,620,000**
**High school**											
E-cigarettes	18.7 (16.1–21.7)	20.4 (17.8–23.4)	23.2 (20.6–25.9)	9.1 (6.7–12.2)	18.9 (15.2–23.4)	12.1 (8.8–16.4)	18.5 (16.1–21.1)	25.1 (19.6–31.5)	14.5 (9.2–22.0)	**19.6 (17.2–22.2)**	**3,020,000**
Cigars	4.7 (3.6–6.1)	5.4 (4.3–6.9)	4.2 (3.2–5.5)	9.2 (7.0–12.1)	5.6 (3.8–8.2)	—^¶^	4.4 (3.6–5.5)	7.2 (4.9–10.4)	6.5 (3.9–10.8)	**5.0 (4.1–6.2)**	**770,000**
Cigarettes	3.9 (2.9–5.2)	5.4 (4.0–7.2)	5.3 (4.0–6.9)	2.8 (1.7–4.6)	4.6 (3.2–6.5)	—^¶^	3.8 (2.8–5.2)	8.0 (5.7–11.2)	7.5 (4.5–12.3)	**4.6 (3.6–6.0)**	**710,000**
Smokeless tobacco	1.4 (1.0–2.0)	4.8 (3.5–6.6)	4.1 (3.0–5.6)	—^¶^	2.2 (1.5–3.2)	—^¶^	3.0 (2.2–4.2)	3.0 (1.8–4.9)	—^¶^	**3.1 (2.3–4.1)**	**480,000**
Hookahs	2.9 (2.1–3.9)	2.6 (1.9–3.4)	1.8 (1.3–2.3)	3.9 (2.5–6.0)	4.4 (2.8–6.9)	—^¶^	2.2 (1.7–2.8)	5.4 (3.8–7.7)	—^¶^	**2.7 (2.1–3.5)**	**420,000**
Heated tobacco products	1.5 (1.1–2.1)	1.3 (0.9–2.0)	1.2 (0.8–1.8)	—^¶^	2.0 (1.4–2.7)	—^¶^	1.0 (0.7–1.5)	3.0 (1.8–4.8)	—^¶^	**1.4 (1.1–1.9)**	**210,000**
Pipe tobacco	0.4 (0.3–0.7)	1.0 (0.6–1.7)	0.9 (0.6–1.5)	—^¶^	—^¶^	—^¶^	—^¶^	—^¶^	—^¶^	**0.7 (0.5–1.1)**	**110,000**
Any tobacco product	22.5 (19.8–25.6)	24.7 (21.6–28.1)	25.9 (23.0–29.2)	18.4 (15.5–21.8)	23.3 (19.4–27.7)	15.7 (12.1–20.2)	22.0 (19.4–24.9)	30.9 (25.3-37.2)	20.4 (14.9-27.2)	**23.6 (21.1-26.4)**	**3,650,000**
Any combustible tobacco product	8.7 (7.1–10.5)	10.2 (8.3–12.3)	8.5 (6.8–10.6)	12.5 (10.3–15.1)	10.7 (8.2–14.0)	6.4 (4.1–9.9)	7.8 (6.5–9.5)	16.2 (12.8–20.2)	13.9 (10.0–19.1)	**9.4 (8.0–11.0)**	**1,450,000**
Multiple tobacco products	7.0 (5.5-8.8)	9.5 (7.5–11.9)	8.9 (7.1–11.0)	6.0 (4.5–8.1)	8.8 (6.4–11.8)	5.9 (3.8–9.0)	7.0 (5.6–8.7)	13.9 (10.6–18.0)	10.8 (6.7–17.0)	**8.2 (6.8**–**10.0)**	**1,270,000**
**Middle school**											
E-cigarettes	4.8 (3.4–6.6)	4.5 (3.5–5.9)	4.3 (3.2–5.6)	2.6 (1.5–4.4)	7.1 (5.2–9.7)	—^¶^	3.8 (2.8–5.1)	12.1 (9.2–15.7)	—^¶^	**4.7 (3.6–6.0)**	**550,000**
Cigars	1.6 (1.1–2.3)	1.4 (1.0–1.9)	0.8 (0.5–1.5)	3.1 (2.2–4.4)	1.8 (1.2–2.9)	—^¶^	1.2 (0.9–1.6)	4.1 (2.4–6.9)	—^¶^	**1.5 (1.2–2.0)**	**180,000**
Cigarettes	2.0 (1.4–2.9)	1.3 (0.9–1.8)	1.3 (0.7–2.2)	2.1 (1.3–3.4)	2.2 (1.5–3.3)	—^¶^	1.2 (0.8–1.9)	5.2 (3.0–8.8)	—^¶^	**1.6 (1.2–2.2)**	**190,000**
Smokeless tobacco	1.0 (0.7–1.5)	1.4 (0.9–2.1)	1.4 (1.0–2.0)	—^¶^	1.0 (0.6–1.7)	—^¶^	0.9 (0.7–1.3)	3.8 (2.3–6.3)	—^¶^	**1.2 (0.9–1.6)**	**140,000**
Hookahs	1.5 (1.0–2.4)	1.2 (0.9–1.7)	0.7 (0.4–1.1)	—^¶^	2.4 (1.4–4.1)	—^¶^	1.1 (0.8–1.6)	3.2 (2.0–5.0)	—^¶^	**1.3 (1.0–1.9)**	**160,000**
Heated tobacco products	1.2 (0.9–1.7)	1.3 (0.8–2.2)	0.9 (0.5–1.4)	—^¶^	2.2 (1.3–3.5)	—^¶^	1.0 (0.6–1.5)	3.5 (2.0–6.1)	—^¶^	**1.3 (0.9–1.8)**	**150,000**
Pipe tobacco	—^¶^	—^¶^	—^¶^	—^¶^	—^¶^	—^¶^	—^¶^	—^¶^	—^¶^	**0.4 (0.2–0.7)**	**40,000**
Any tobacco product	6.8 (5.3–8.8)	6.6 (5.3–8.1)	5.7 (4.6–7.2)	6.7 (5.1–8.8)	9.4 (7.3–12.0)	—^¶^	5.5 (4.4–6.9)	16.5 (13.0–20.5)	6.4 (4.0–9.9)	**6.7 (5.5**–**8.2)**	**800,000**
Any combustible tobacco product	3.8 (3.0–5.0)	2.9 (2.2–3.8)	2.1 (1.4–3.2)	5.0 (3.6–6.7)	4.8 (3.4–6.7)	—^¶^	2.6 (2.0–3.5)	9.0 (6.1–13.0)	3.3 (1.8–5.8)	**3.4 (2.7–4.2)**	**400,000**
Multiple tobacco products	3.1 (2.3–4.1)	2.6 (2.0–3.5)	2.2 (1.5–3.1)	3.3 (2.2–5.0)	4.0 (2.7–5.9)	—^¶^	2.1 (1.6–2.9)	8.2 (5.8–11.7)	—^¶^	**2.8 (2.2**–**3.7)**	**340,000**

**Abbreviation:** CI = confidence interval.

* Past 30-day use of e-cigarettes was determined by asking “During the past 30 days, on how many days did you use e-cigarettes?” Past 30-day use of cigarettes was determined by asking “During the past 30 days, on how many days did you smoke cigarettes?” Past 30-day use of cigars was determined by asking “During the past 30 days, on how many days did you smoke cigars, cigarillos, or little cigars?” Smokeless tobacco was defined as use of chewing tobacco, snuff, dip, snus, or dissolvable tobacco products. Past 30-day use of smokeless tobacco was determined by asking the following question for use of chewing tobacco, snuff, and dip: “During the past 30 days, on how many days did you use chewing tobacco, snuff, or dip?” and the following question for use of snus and dissolvable tobacco products: “In the past 30 days, which of the following products did you use on at least one day?” Responses from these questions were combined to derive overall smokeless tobacco use. Past 30-day use of hookahs was determined by asking “During the past 30 days, on how many days did you smoke tobacco in a hookah or waterpipe?” Past 30-day use of pipe tobacco (not hookahs) was determined by asking “In the past 30 days, which of the following products have you used on at least one day?” Past 30-day use of heated tobacco products was determined by asking “During the past 30 days, on how many days did you use heated tobacco products?” Because of missing data on the past 30-day use questions, denominators for each tobacco product might be different.

^†^ Hispanic persons could be of any race (White; Black or African American; or other race [i.e., American Indian or Alaska Native; Asian; Hawaiian or other Pacific Islander]).

^§^ Estimated weighted total number of current tobacco product users was rounded down to the nearest 10,000 persons. Overall estimates were reported among 14,531 U.S. middle and high school students. School level was determined by self-reported grade level: high school (grades 9–12; n = 7,453) and middle school (grades 6–8; n = 7,042). Overall population estimates might not directly total to sums of corresponding subgroup population estimates because of rounding or inclusion of students who did not self-report sex, race/ethnicity, sexual identity, or grade level.

^¶^ Data were statistically unreliable because of unweighted denominator <50 or a relative standard error >30%.

** In 2020, any tobacco product use was defined as use of any tobacco product (e-cigarettes, cigarettes, cigars, smokeless tobacco, hookahs, pipe tobacco, bidis [small brown cigarettes wrapped in a leaf], or heated tobacco products) on ≥1 day during the past 30 days.

^††^ Any combustible tobacco product use was defined as use of cigarettes, cigars, hookahs, pipe tobacco, or bidis on ≥1 day during the past 30 days.

^§§^ In 2020, multiple tobacco product use was defined as use of two or more tobacco products (e-cigarettes, cigarettes, cigars, smokeless tobacco, hookahs, pipe tobacco, bidis, or heated tobacco products) on ≥1 day during the past 30 days.

Among middle school students, 6.7% (800,000) reported current use of any tobacco product, 3.4% (400,000; 50.7% of any tobacco product users) reported current use of any combustible tobacco product, and 2.8% (340,000; 41.8% of any tobacco product users) reported current use of multiple tobacco products. By type of product, current use among middle school students was highest for e-cigarettes (4.7%), followed by cigarettes (1.6%), cigars (1.5%), hookahs (1.3%), heated tobacco products (1.3%), smokeless tobacco (1.2%), and pipe tobacco (0.4%). Among middle school students, any tobacco product use was reported by 6.8% of females and 6.6% of males; by 9.4% of Hispanic, 6.7% of non-Hispanic Black, and 5.7% of non-Hispanic White students; and by 16.5% of those identifying as lesbian, gay, or bisexual, 5.5% of those identifying as heterosexual, and 6.4% of those reporting “not sure” about their sexual identity.

From 2019 to 2020, among high school (Figure 1) and middle school students (Figure 2), significant declines (p<0.05) occurred in current use of any tobacco product (high school: 31.2% to 23.6%, RPC = −24.4%; middle school: 12.5% to 6.7%, RPC = −46.4%), any combustible tobacco product (high school: 12.0% to 9.4%, RPC = −21.7%; middle school: 4.8% to 3.4%, RPC = −29.2%), multiple tobacco products (high school: 10.8% to 8.2%, RPC = −24.1%; middle school: 4.0% to 2.8%, RPC = −30.0%), e-cigarettes (high school: 27.5% to 19.6%, RPC = −28.7%; middle school: 10.5% to 4.7%, RPC = −55.2%), cigars (high school: 7.6% to 5.0%, RPC = −34.2%; middle school: 2.3% to 1.5%, RPC = −34.8%), and smokeless tobacco (high school: 4.8% to 3.1%, RPC = −35.4%; middle school: 1.8% to 1.2%, RPC = −33.3%). During 2019–2010, no significant change in current use of cigarettes, hookahs, pipe tobacco, or heated tobacco products occurred among high or middle school students.

**FIGURE 1:**
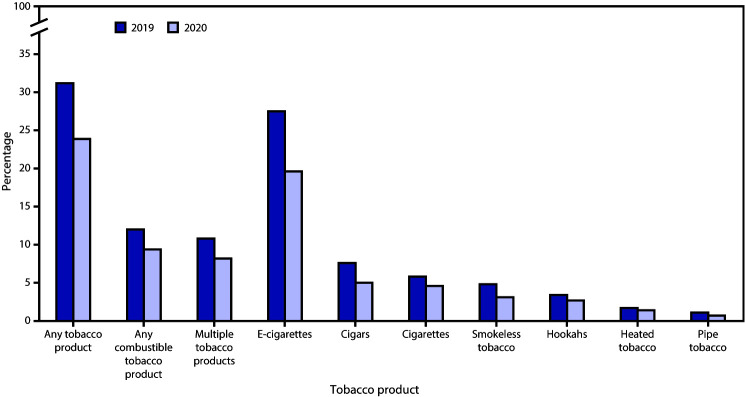
Percentage of current use of selected tobacco products,* any tobacco product,^†^ any combustible tobacco product,^§^ and multiple tobacco products ^¶^ among high school students — National Youth Tobacco Survey, United States, 2019 and 2020** * Current use is defined as use on ≥1 day during the past 30 days for each product. ^†^ In 2020, any tobacco product use was defined as use of any tobacco product (e-cigarettes, cigarettes, cigars, smokeless tobacco, hookahs, pipe tobacco, bidis [small brown cigarettes wrapped in a leaf], or heated tobacco products) on ≥1 day during the past 30 days. In 2019, consistent with previously published estimates, any tobacco product use was defined as use of any tobacco product (e-cigarettes, cigarettes, cigars, smokeless tobacco, hookahs, pipe tobacco, or bidis) on ≥1 day during the past 30 days. ^§^ Any combustible tobacco product use was defined as use of cigarettes, cigars, hookahs, pipe tobacco, or bidis on ≥1 day during the past 30 days. ^¶^ In 2020, multiple tobacco product use was defined as use of two or more tobacco products (e-cigarettes, cigarettes, cigars, smokeless tobacco, hookahs, pipe tobacco, bidis, or heated tobacco products) on ≥1 day during the past 30 days. In 2019, consistent with previously published estimates, multiple tobacco product use was defined as use of two or more tobacco products (e-cigarettes, cigarettes, cigars, smokeless tobacco, hookahs, pipe tobacco, or bidis) on ≥1 day during the past 30 days. ** During 2019–2020, significant declines in the use of any tobacco product (p<0.001), any combustible tobacco product (p = 0.018), multiple tobacco products (p = 0.020), e-cigarettes (p<0.001), cigars (p<0.001), and smokeless tobacco (p = 0.031) were observed. No significant change in use of cigarettes, hookah, heated tobacco products, or pipe tobacco occurred.

**FIGURE 2:**
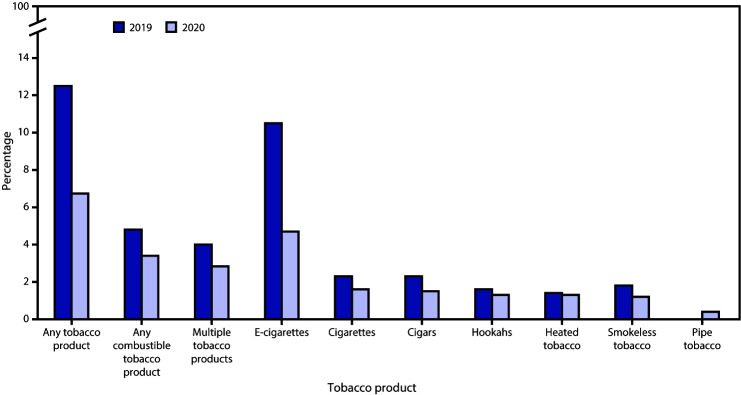
Percentage of current use of selected tobacco products,*,^†^ any tobacco product,^§^ any combustible tobacco product,^¶^ and multiple tobacco products** among middle school students — National Youth Tobacco Survey, United States, 2019 and 2020^††^ * Current use is defined as use on ≥1 day during the past 30 days for each product. ^†^ Estimate for “pipe tobacco, 2019” is suppressed because of relative standard error >30% or unweighted denominator <50. ^§^ In 2020, any tobacco product use was defined as use of any tobacco product (e-cigarettes, cigarettes, cigars, smokeless tobacco, hookahs, pipe tobacco, bidis [small brown cigarettes wrapped in a leaf], or heated tobacco products) on ≥1 day during the past 30 days. In 2019, consistent with previously published estimates, any tobacco product use was defined as use of any tobacco product (e-cigarettes, cigarettes, cigars, smokeless tobacco, hookahs, pipe tobacco, or bidis) on ≥1 day during the past 30 days. ^¶^ Any combustible tobacco product use was defined as use of cigarettes, cigars, hookahs, pipe tobacco, or bidis on ≥1 day during the past 30 days. ** In 2020, multiple tobacco product use was defined as use of two or more tobacco products (e-cigarettes, cigarettes, cigars, smokeless tobacco, hookahs, pipe tobacco, bidis, or heated tobacco products) on ≥1 day during the past 30 days. In 2019, consistent with previously published estimates, multiple tobacco product use was defined as use of two or more tobacco products (e-cigarettes, cigarettes, cigars, smokeless tobacco, hookahs, pipe tobacco, or bidis) on ≥1 day during the past 30 days. ^††^ During 2019–2020, significant declines in the use of any tobacco product (p<0.001), any combustible tobacco product (p = 0.013), multiple tobacco products (p = 0.025), e-cigarettes (p<0.001), cigars (p = 0.012), and smokeless tobacco (p = 0.038) were observed. No significant change in use of cigarettes, hookahs, or heated tobacco products occurred. Because of the suppression of the pipe tobacco estimate in 2019, no comparison was made during 2019–2020.

## Discussion

Use of any tobacco product by youths declined by an estimated 1.73 million from 6.20 million in 2019 (*3*) to 4.47 million in 2020. Despite this decline, in 2020 nearly one in four U.S. high school students and approximately one in 15 middle school students still reported current use of any tobacco product. Continued efforts are warranted to sustain this progress and to prevent and reduce all forms of tobacco product use among U.S. youths (*1*,*2*).

Among both middle and high school students, current use of e-cigarettes declined from 2019 to 2020, reversing previous trends and returning current e-cigarette use to levels similar to those observed in 2018 (*4*). Declines in current cigar smoking and smokeless tobacco product use also occurred, as did youths’ use of any combustible tobacco products and multiple tobacco products. Together, these changes contributed to an overall reduction in any tobacco product use by youths during 2019–2020. These declines were likely attributable to multiple factors at the national, state, and local level. For example, in December 2019, the federal minimum age of sale of all tobacco product types increased from 18 to 21 years (*5*). Under the authority of the 2009 Family Smoking Prevention and Tobacco Control Act, FDA issued guidance in January 2020 to prioritize enforcement against certain flavored e-cigarette products that appeal to youths, including mint and fruit flavors (*6*). Several states and communities also recently restricted the sale of flavored tobacco products, including e-cigarettes.^§§§^ In addition, public health efforts to address the multistate outbreak of e-cigarette, or vaping, product use–associated lung injury (EVALI) might have contributed to these declines in youth e-cigarette use (*7*). Furthermore, targeted actions to address the youth e-cigarette epidemic occurred, including FDA’s public education campaign to reduce youth e-cigarette, smokeless tobacco, and cigarette use.^¶¶¶^

Despite declines in youths’ use of combustible tobacco products since 2011 (*4*), no change in current cigarette smoking occurred during 2019–2020. Among all students who currently used any tobacco product, approximately 42% (1.87 million) reported smoking combustible tobacco products in 2020. However, a decline in current cigar smoking did occur during 2019–2020. Continued actions are warranted to help ensure sustained progress in preventing and reducing youths’ use of all forms of tobacco products, including those that are combustible, noncombustible, and electronic.

The findings in this report are subject to at least three limitations. First, the data collection period was truncated because of the coronavirus disease 2019 pandemic, resulting in a lower school participation rate (49.9%) compared with recent NYTS cycles (average across 2011–2019 NYTS cycles = 78.2%). However, the 2020 NYTS student participation rate (87.4%) was high, and the weighted sample yielded nationally representative estimates.**** Second, these data were self-reported and might be subject to recall and response biases. Finally, these findings might not be generalizable to youths who are homeschooled, have dropped out of school, are in detention centers, or are enrolled in alternative schools.

In 2020, approximately one in six U.S. middle and high school students, or approximately 4.47 million youths overall, reported current use of any tobacco product. The comprehensive and sustained implementation of evidence-based tobacco control strategies at the national, state, and local levels, combined with tobacco product regulation by FDA, is warranted for continuing progress toward reducing and preventing all forms of tobacco product use among U.S. youths. Such strategies include increasing prices of tobacco products, protecting persons from exposure to secondhand smoke and e-cigarette aerosol, sustaining hard-hitting media campaigns that warn about the dangers of tobacco product use, restricting youth access to tobacco products, prohibiting the sale of all flavored tobacco products, and development of regulations to reduce youth appeal and addictiveness of tobacco products (*1*–*3*,*8*–*10*). In addition, as the tobacco product landscape continues to diversify, surveillance for all forms of tobacco product use, including novel products, by youths is important to inform public health policy and practice at the local, state, and national levels.

**Summary**

**What is already known?**

Tobacco use is the leading cause of preventable disease and death in the United States; nearly all tobacco use begins during youth and young adulthood.

**What is added by this report?**

In 2020, 23.6% (3.65 million) of high school and 6.7% (800,000) of middle school students reported current (past 30-day) use of any tobacco product. From 2019 to 2020, decreases among high school and middle school students occurred in current use of any tobacco product, combustible tobacco products, multiple tobacco products, e-cigarettes, cigars, and smokeless tobacco.

**What are the implications for public health?**

The comprehensive and sustained implementation of evidence-based tobacco control strategies, combined with tobacco product regulation by the Food and Drug Administration, is warranted to help sustain this progress and prevent and reduce all forms of tobacco product use among U.S. youths.

## Footnotes

* The survey was programmed using an application that did not require Internet access for use. Eligible students who were absent on the day of survey administration could participate in the NYTS using a web-based make-up survey.

† The data collection timeline was truncated because of widespread school closures during the coronavirus disease 2019 pandemic; data collection was anticipated to occur through May 2020. For comparison, the 2019 NYTS data were collected during February 15–May 24, 2019.

§ https://www.cdc.gov/tobacco/data_statistics/surveys/nyts/index.htm.

¶ Definition of smokeless tobacco includes chewing tobacco, snuff, or dip; snus; and dissolvable tobacco. Use of individual smokeless tobacco products is not reported.

** Use of pipe tobacco was assessed among respondents who reported ever use of one or more “other tobacco product” by the question “In the past 30 days, which of the following products have you used on at least one day? (Select one or more)” Respondents could select tobacco product(s) they had used from the list: roll-your-own cigarettes; pipes filled with tobacco (not hookah or waterpipe); snus; dissolvable tobacco products; bidis. Estimates of current use of roll-your-own cigarettes and bidis are not reported.

†† Respondents were first asked about heated tobacco product use in 2019. Questions assessing awareness, ever use, and current use of heated tobacco products were accompanied by a brief description: “The next section is about heated tobacco products. Some persons refer to these products as “heat-not-burn” tobacco products. Heated tobacco products heat tobacco sticks or capsules to produce a vapor. They are different from e-cigarettes, which heat a liquid to produce a vapor. Some brands of heated tobacco products include iQOS, glo, and Eclipse.”

§§ In 2020, any tobacco product use is defined as current use of one or more of the following tobacco products on ≥1 day during the past 30 days: e-cigarettes, cigarettes, cigars, smokeless tobacco, hookahs, pipe tobacco, bidis, or heated tobacco products. In 2019, consistent with previously published estimates, any tobacco product use is defined as current use of one or more of the following tobacco products on ≥1 day during the past 30 days: e-cigarettes, cigarettes, cigars, smokeless tobacco, hookahs, pipe tobacco, or bidis. In 2020, inclusion of heated tobacco products did not significantly change overall estimates of any current tobacco product use among youths.

¶¶ In 2019 and 2020, combustible tobacco product use is defined as current use of one or more of the following tobacco products on ≥1 day during the past 30 days: cigarettes, cigars, hookahs, pipe tobacco, or bidis.

*** In 2020, multiple tobacco product use is defined as current use of two or more of the following tobacco products on ≥1 day during the past 30 days: e-cigarettes, cigarettes, cigars, smokeless tobacco, hookahs, pipe tobacco, bidis, or heated tobacco products. In 2019, consistent with previously published estimates, multiple tobacco product use is defined as current use of two or more of the following tobacco products on ≥1 day during the past 30 days: e-cigarettes, cigarettes, cigars, smokeless tobacco, hookahs, pipe tobacco, or bidis. In 2020, inclusion of heated tobacco products did not change overall estimates of multiple tobacco product use among youth significantly.

††† Data were weighted to account for the complex survey design and to adjust for nonresponse. Population estimates of current use were rounded down to the nearest 10,000 persons.

§§§ https://www.tobaccofreekids.org/assets/factsheets/0398.pdf.

¶¶¶ https://www.fda.gov/tobacco-products/public-health-education/youth-and-tobacco.

**** In addition to standard nonresponse bias analysis assessing differences in responding and nonresponding schools, for the 2020 cycle, extended nonresponse analyses were conducted to examine differences between participating schools (180) and schools that were recruited, but did not participate (74) because of widespread school closures during the coronavirus disease 2019 pandemic. These groups of schools did not differ by U.S. Census region. School participation was significantly higher among nonurban schools and those with a lower proportion of the student population that was non-Hispanic Black. However, both of these indicators were used in the creation of survey weight adjustments to mitigate potential biases.

## References

[1] US Department of Health and Human Services. The health consequences of smoking—50 years of progress. Atlanta, GA: US Department of Health and Human Services, CDC; 2014. https://www.ncbi.nlm.nih.gov/books/NBK179276/pdf/Bookshelf_NBK179276.pdf

[2] US Department of Health and Human Services. Preventing tobacco use among youth and young adults. Atlanta, GA: US Department of Health and Human Services, CDC; 2012. https://www.cdc.gov/tobacco/data_statistics/sgr/2012/index.htm

[3] Wang TW, Gentzke AS, Creamer MR, Tobacco product use and associated factors among middle and high school students—United States, 2019. MMWR Surveill Summ 2019;68(No. SS-12). 10.15585/mmwr.ss6812a1 31805035 PMC6903396

[4] Gentzke AS, Creamer M, Cullen KA, Vital signs: tobacco product use among middle and high school students—United States, 2011–2018. MMWR Morb Mortal Wkly Rep 2019;68:157–64. 10.15585/mmwr.mm6806e1 30763302 PMC6375658

[5] Food and Drug Administration. newly signed legislation raises federal minimum age of sale of tobacco products to 21. Silver Spring, MD: US Department of Health and Human Services, Food and Drug Administration; 2019. https://www.fda.gov/tobacco-products/ctp-newsroom/newly-signed-legislation-raises-federal-minimum-age-sale-tobacco-products-21

[6] Center for Tobacco Products. Enforcement priorities for Electronic Nicotine Delivery Systems (ENDS) and other deemed products on the market without premarket authorization (revised). Silver Spring, MD: US Department of Health and Human Services, Food and Drug Administration; 2020. https://www.fda.gov/media/133880/download

[7] King BA, Jones CM, Baldwin GT, Briss PA. The EVALI and youth vaping epidemics—implications for public health. N Engl J Med 2020;382:689–91. 10.1056/NEJMp1916171 31951683 PMC7122126

[8] US Department of Health and Human Services. E-cigarette use among youth and young adults: a report of the Surgeon General. Atlanta, GA: US Department of Health and Human Services, CDC; 2016. https://www.cdc.gov/tobacco/data_statistics/sgr/e-cigarettes/pdfs/2016_sgr_entire_report_508.pdf

[9] US Department of Health and Human Services. Surgeon General’s advisory on e-cigarette use among youth. Rockville, MD: US Department of Health and Human Services, Office of the Surgeon General; 2018. https://e-cigarettes.surgeongeneral.gov/documents/surgeon-generals-advisory-on-e-cigarette-use-among-youth-2018.pdf

[10] CDC. Best practices for comprehensive tobacco control programs—2014. Atlanta, GA: US Department of Health and Human Services, CDC; 2014. https://www.cdc.gov/tobacco/stateandcommunity/best_practices/index.htm

